# Species Adaptive Strategies and Leaf Economic Relationships across Serpentine and Non-Serpentine Habitats on Lesbos, Eastern Mediterranean

**DOI:** 10.1371/journal.pone.0096034

**Published:** 2014-05-06

**Authors:** George C. Adamidis, Elena Kazakou, Nikolaos M. Fyllas, Panayiotis G. Dimitrakopoulos

**Affiliations:** 1 Biodiversity Conservation Laboratory, Department of Environment, University of the Aegean, Mytilene, Greece; 2 Montpellier SupAgro, UMR Centre d'Ecologie Fonctionnelle et Evolutive, CNRS, UMR 5175, Montpellier, France; 3 Department of Ecology & Systematics, Faculty of Biology, University of Athens, Athens, Greece; Duke University, United States of America

## Abstract

Shifts in species' traits across contrasting environments have the potential to influence ecosystem functioning. Plant communities on unusually harsh soils may have unique responses to environmental change, through the mediating role of functional plant traits. We conducted a field study comparing eight functional leaf traits of seventeen common species located on both serpentine and non-serpentine environments on Lesbos Island, in the eastern Mediterranean. We focused on species' adaptive strategies across the two contrasting environments and investigated the effect of trait variation on the robustness of core ‘leaf economic’ relationships across local environmental variability. Our results showed that the same species followed a conservative strategy on serpentine substrates and an exploitative strategy on non-serpentine ones, consistent with the leaf economic spectrum predictions. Although considerable species-specific trait variability emerged, the single-trait responses across contrasting environments were generally consistent. However, multivariate-trait responses were diverse. Finally, we found that the strength of relationships between core ‘leaf economic’ traits altered across local environmental variability. Our results highlight the divergent trait evolution on serpentine and non-serpentine communities and reinforce other findings presenting species-specific responses to environmental variation.

## Introduction

Plants growing on special substrates (e.g. serpentine, limestone, gypsum, dolomite and shale) attract a lot of attention, not only due to their major contribution to global biodiversity but also because of their unique ecological character that may induce diverse community responses to environmental change [1]. Shifts in species traits across contrasting environments (e.g. productive vs unproductive, polluted vs unpolluted) reveal the alternative strategies of plants for reproductive success and survival [2] and have the potential to influence ecosystem functioning (reviewed in [3], [4]). For example, the harsh conditions of a substrate may limit the range of ecological strategies and thus filter the available species pools leading to communities dominated by species with similar functional traits [5], [6]. Thus, dry environmental conditions may select for species with traits that allow them to use nutrient and water resources more conservatively [7]. However, although within each habitat abiotic environment leads to trait convergence by selecting similar trait values between coexisting species [8], niche differentiation leads to limiting similarity of trait values (trait divergence) [9]. In this context, plant communities on special substrates may be relatively responsive to changes (e.g. changes in rainfall, nitrogen deposition) due to their multiple limitations [10]. On the other hand, plants growing on special soils may be especially resistant to environmental changes due to their adaptations to harsh conditions [11]. Although shifts in functional traits across contrasting environments (e.g. wet-dry, productive-unproductive, etc.) may be expected [12] and have been captured by the major leaf economic dimensions [13], [14], [15], the variation of fundamental leaf traits across habitats and/or within species is important to consider [16], [17]. Moreover, although the relationships demonstrated by the leaf economic spectrum are robust at the global scale, the importance of trait variability on its core relationships is not well documented across locally contrasting environments [17].

Serpentine substrates are a well known example of a harsh environment for plants [18], [19], [20] and constitute efficient model systems for investigating variation on plant functional traits. Furthermore, serpentine ecosystems are important reservoirs for biodiversity as their flora includes a high number of rare and endemic species that present morphological and physiological adaptations to extreme conditions [18]. In a recent study, Californian serpentine grasslands showed greater resistance to environmental (climatic) fluctuation relative to non-serpentine [21], due to the presence of species with enhanced stress-tolerance traits (e.g. slow growth-rate, low height, low specific leaf area, high root/shoot biomass quotient; [22]). Mechanisms like abiotic stress and patchiness may also explain the greater temporal stability (greater resistance to environmental fluctuations) of plant communities established in harsh environments [21], [22], [23], [24], [25].

Serpentine plant communities on Lesbos Island (eastern Mediterranean) have also shown higher short-term temporal stability in terms of species composition, relative to non-serpentine ones [26]. Although this may suggest a possible conservative response of these communities to environmental fluctuations (e.g. climate change [23], [24]), it is not yet known if the higher short-term stability corresponds to species traits associated with efficient resource conservation. In this study we focus on species' adaptive strategies across serpentine and non-serpentine habitats on Lesbos and test across local contrasting environments the effect of trait variation on: a) the predictability of trait responses and b) the repeatability of relationships between core ‘leaf economic’ traits. Specifically, the following three questions are addressed: (1) Do species occurring on both serpentine and non-serpentine substrates present different adaptive strategies in response to different substrate types? If there is significant species leaf trait differentiation between the two contrasting substrates, will species occurring on serpentine substrates tend to have traits that allow them efficient resource conservation and species occurring on non-serpentine substrates tend to acquire resources rapidly? (2) Is there a repeatable ranking of species based on their leaf traits across different substrates? And finally, (3) if there is significant trait variation in response to substrate differentiation, are the relationships between the traits of the leaf economic spectrum conserved across contrasting substrates?

## Materials and Methods

### Ethics statement

No specific permits and/or approvals were required for the described study sites on Lesbos (Greece). Given that all the localities selected for our sampling are owned and managed by the government and are not private property or protected, no specific permits were required. In addition, our field study did not include any endangered or protected plant species. All data included in this study are freely available upon request.

### Study sites

The study was conducted between May and June 2008 at four sites (Vatera, Ampeliko, Olympos and Loutra) located in the central and south-eastern part of Lesbos. All sites were dominated by herbaceous vegetation. A serpentine and an adjacent non-serpentine locality were chosen for comparisons in each of the four sites. The serpentine localities were selected based on how well they represented the altitudinal and geographic range of serpentine habitats on the island and on the availability of accessible and adjacent non-serpentine areas within close proximity (0.6–7 km) and similar disturbance history and climatic conditions. All selected non-serpentine localities were located on alluvial plains. A detailed description of the sites is available in Adamidis et al. [26] and in Kazakou et al. [27].

### Leaf trait measurements

We selected seventeen herbaceous species (Table S1) that were sufficiently common and broadly distributed, all located in the two contrasting substrates. In order to capture the trait syndromes and thus the ecological strategies of these species, eight leaf traits (Table 1) were measured on the youngest fully expanded leaves with 10 replicates per species using standardized procedures [28]. Leaf length (LL) was measured as the distance from the leaf tip to the point of attachment with the stalk. Leaf width (LW) was measured as the diameter of the maximum imaginary circle fitted within the leaf [29]. Specific leaf area (SLA) was calculated as the ratio of the water-saturated leaf area to the leaf dry mass. Leaf dry matter content (LDMC) was determined as the ratio of leaf dry mass to water-saturated fresh mass. Leaf thickness (LT) was estimated by the (SLA x LDMC)^−1^ product, or the water-saturated leaf fresh mass to leaf area ratio [30]. Leaf material was pooled to produce three batches that were ground separately and their leaf nitrogen (LNC) and leaf carbon (LCC) content were measured using an elemental analyser (Carlo Erba Instruments, model EA 1108, Milan, Italy). Data for leaf phosphorus concentration were obtained from Kazakou et al. [27].

**Table 1:** Leaf trait abbreviations and units.

Leaf trait	Abbreviation	Unit
Specific leaf area	SLA	m^2^ kg^−1^
Leaf dry matter content	LDMC	mg g^−1^
Leaf thickness	LT	µm
Leaf length	LL	cm
Leaf width	LW	cm
Leaf nitrogen per mass	LNC	mg g^−1^
Leaf carbon per mass	LCC	mg g^−1^
Leaf phosphorus concentration	LPC	mg g^−1^

### Data analysis

Where necessary, leaf traits were transformed to their natural logarithms before analysis to improve normality and homoscedasticity. Analyses of variance assuming species and substrate as fixed factors and locality as a random factor were used to explore the effects of species and substrate on all measured leaf traits. In the case of LPC, locality was not included in the ANOVA due to the sampling design and thus a two way ANOVA with both species and substrate as fixed factors was conducted. Spearman rank correlation coefficients were calculated on untransformed leaf trait values to test whether the rank of species responses for each given trait was conserved across substrate types. For the evaluation of these bivariate correlations the species mean trait values were used for each substrate type (e.g. LDMC on serpentine substrates vs. non-serpentine). This analysis assesses the consistency of species ranking across different substrates based on species single-trait responses, i.e the species hierarchy in terms of mean trait values. To examine whether species shifts in response to different substrates were consistent across the multivariate-trait space a PCA ordination of the z-transformed species mean trait values was used for each substrate type. This analysis reduced our dimensionality in two axes (74% and 76% of the total variance explained for septenine and non-serpentine substrates respectively) and thus the position of each variable in two dimensions. Each PCA produced a set of two-dimensional coordinates for each species-by-substrate combination, describing the position of each species on the multivariate-trait space for each substrate. In order to graphically represent the shifts of species leaf traits across multivariate trait space in response to substrate type differentiation, the coordinates of each species-by-substrate combination were subtracted from each species coordinates on the serpentine substrate. In this way, the coordinates of each species on the multivariate-trait space for serpentine substrates are represented by the origin of the axes. Pearson correlation coefficients were used for the investigation of among-trait relationships within each substrate type.

Standardized major axis (SMA) analysis was used to examine whether relationships between core ‘leaf economic’ traits were conserved across different substrates. SLA-LNC, SLA-LPC, LPC-LNC and SLA-LDMC relationships were quantified and compared across different substrates. SMA was used to test whether these bivariate relationships differed in slope and when no significant differentiation emerged, differences in intercept (elevation) or position along a common slope were tested. All the previous statistical analyses were carried out using the R statistical platform [31].

## Results

### Effects of substrate type and species on leaf traits

The ANOVA revealed significant effect of ‘substrate’ on all measured leaf traits (*P*<0.05; Table 2) except LNC and LPC (*P*>0.05). On average, SLA, LL and LW were respectively 20%, 15.4% and 30% higher for individuals occurring on non-serpentine substrates while LDMC and LT were respectively 7.8% and 22% higher for individuals occurring on serpentine substrates (Table 2). There were also significant ‘substrate x species’ (*P*<0.05; Table 2) interactions for all traits (apart from LCC), indicating that differences among species and localities affected the response of leaf traits to substrate types.

**Table 2:** Results of ANOVA (*F-*values, probabilities and R-squared) for the effects of substrate, species and their interaction on leaf traits.

Source	df	SLA	LDMC	LT	LL	LW	df	LNC	LCC	df	LPC
Species	16	25.25***	153.09***	61.31***	161.27***	152.70***	16	7.53***	3.593***	16	13.956***
Substrate	1	100.24***	31.32***	103.64***	68.94***	114.44***	1	0.194 ^NS^	0.739 ^NS^	1	0.047 ^NS^
Species x Substrate	16	9.56***	8.18***	14.70***	7.57***	5.56***	16	2.483***	1.038 ^NS^	16	4.962***
Residuals	950						182			119	
R squared		0.410	0.733	0.583	0.744	0.735		0.468	0.291		0.701
Differences across substrate types		Non-serp > Serp	Serp > Non-serp	Serp > Non-serp	Non-serp > Serp	Non-serp > Serp		-	-		-

***, *P*<0.0001; **, *P*<0.01; *, *P*<0.05; ^NS^, not significant. Abbreviations in Table 1.

Differences between serpentine (*Serp*) and non-serpentine (*Non-serp*) substrate types are presented.

### Consistency of species' single- and multivariate- trait responses across different substrates

**Figure 1:**
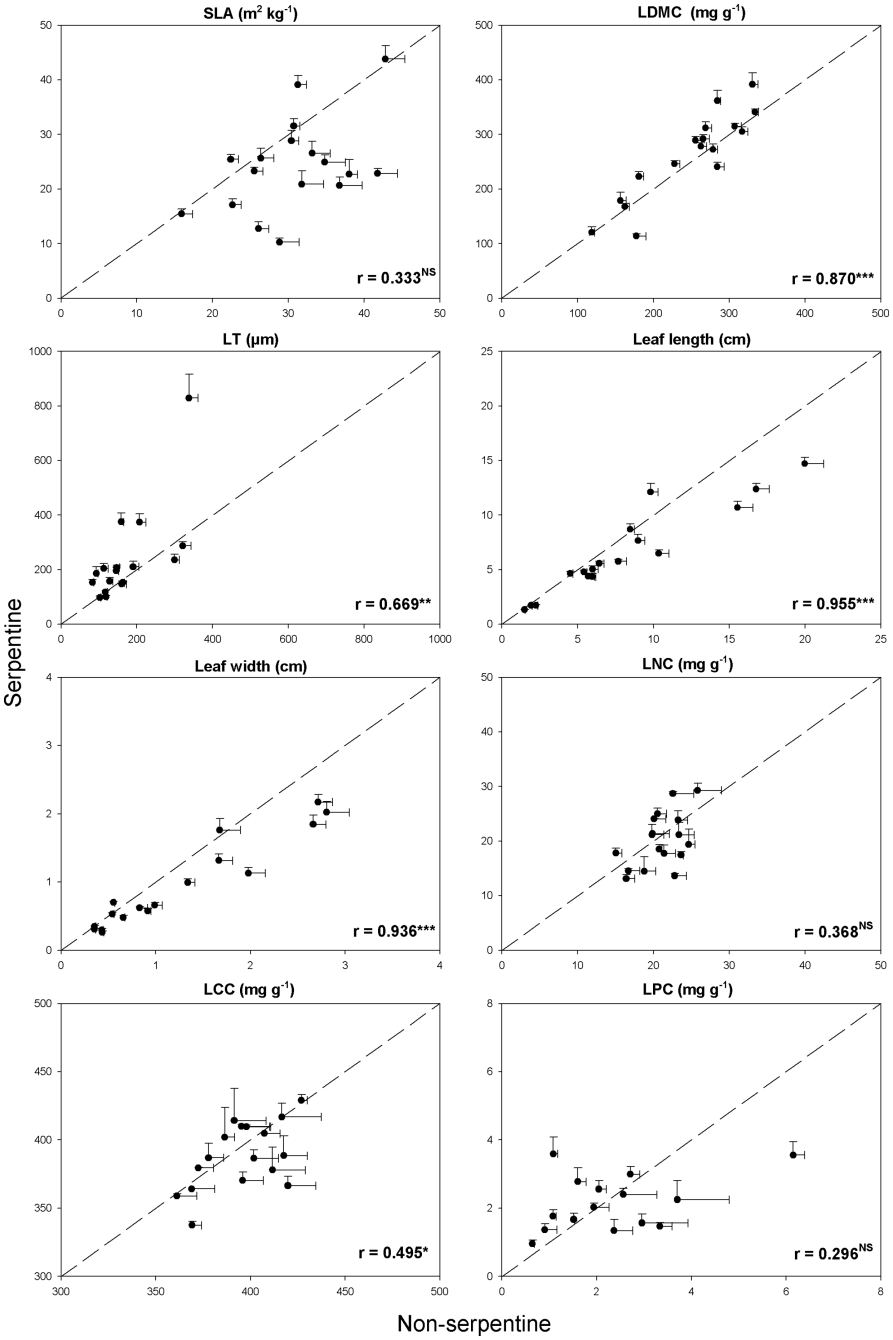
Response patterns of SLA, LDMC, LT, LL, LW, LNC, LCC and LPC to different substrate types for seventeen herbaceous species (solid circles). Spearman rank correlation coefficients are given: ***, *P*<0.0001;**, *P*<0.01; *, *P*<0.05; ^NS^, not significant. The dotted line represents the 1∶1 line. The bi-directional bars represent the standard error of means. Abbreviations are given in Table 1.

Although the significant ‘substrate x species’ interactions denote that not all species respond similarly to substrate type transition, further analysis revealed significant across-species-correlations in mean values of leaf traits between the two contrasting substrates. Species presented a consisting ranking between serpentine and non-serpentine substrates for all leaf traits except for SLA, LNC and LPC (*P*>0.05) (Figure 1).

**Figure 2:**
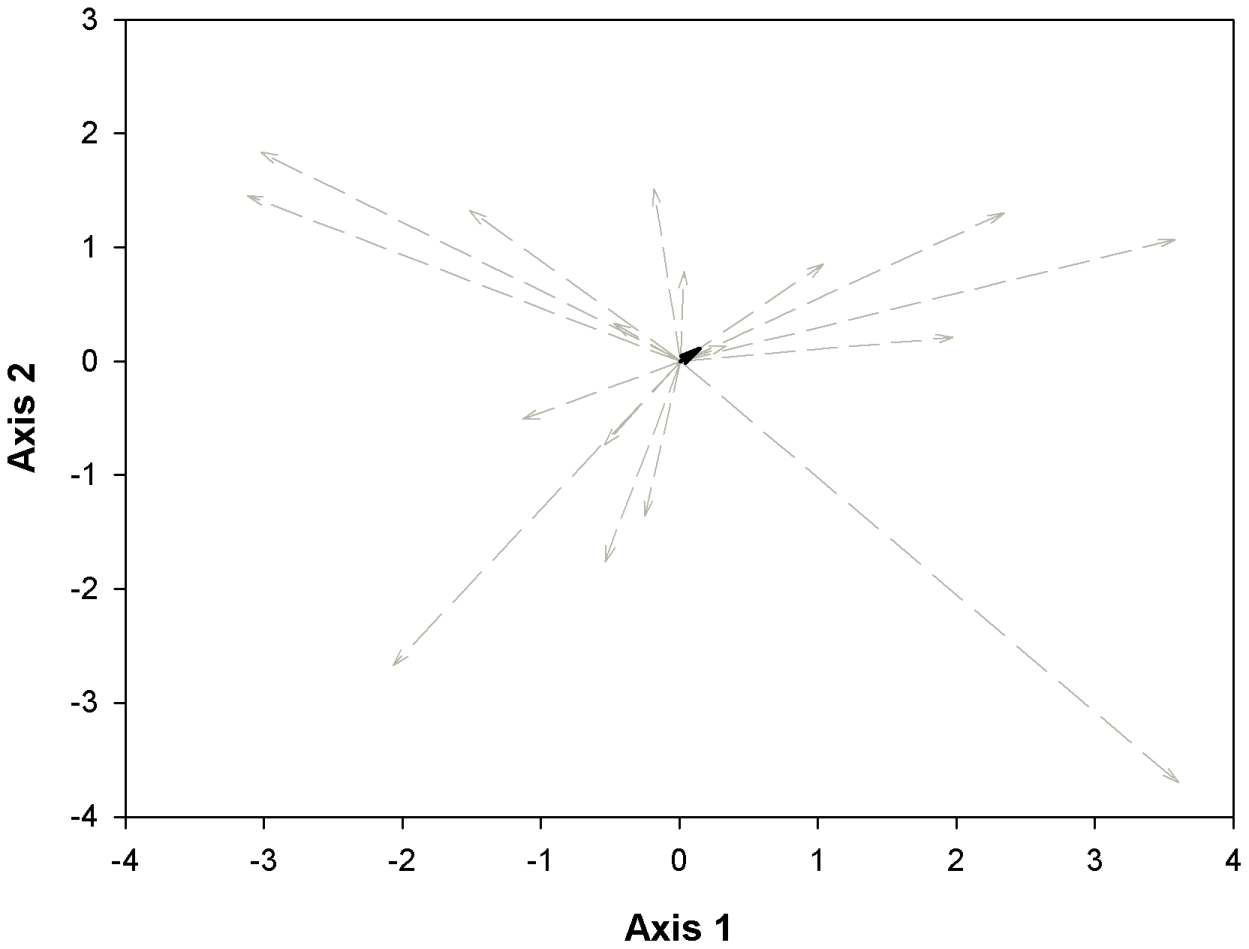
Shifts of species leaf traits across multivariate trait space in response to substrate type differentiation. The grey dashed zero-centered vectors represent the species' shifts connecting the position of each species in leaf trait space across the two different substrate types. The black vector represents the mean shift of all species.

On the other hand, the PCA of the z-transformed species mean trait values showed significant variation in species shifts across multivariate-trait space in response to substrate differentiation. The transition from serpentine to non-serpentine substrates caused widely diverse shifts in species' leaf trait values (Figure 2) that resulted in a non-significant mean shift along the two dimensions (mean ±95% CI; Axis 1: 0.0059±1.03; Axis 2: 0.0059±0.82).

### Correlations among leaf traits and their consistency across different substrates

SLA was negatively correlated with both LDMC and LT and positively correlated with LL, LW, LNC and LPC in the two substrate types (Table 3). SLA and LCC were positively correlated only on non-serpentine substrates (Table 3). LDMC was negatively correlated with LT and LPC and positively correlated with LL on both substrates. On serpentine substrates a negative correlation between LDMC and LW emerged. LL was positively correlated with LPC on serpentine substrates and negatively correlated with LT and LW on both substrates. LW was positively correlated with LNC on serpentine substrates and with LPC on non-serpentine substrates. Finally LNC was positively correlated with LCC on non-serpentine substrates and with LPC on both substrates (Table 3).

**Table 3:** Pearson correlation coefficients between measured leaf traits within each substrate type.

Leaf trait	Substrate type	LDMC	LT	LL	LW	LNC	LCC	LPC
SLA	non-serpentine	−0.09*	−0.61***	0.17***	0.07*	0.21**	0.17*	0.69***
	serpentine	−0.16***	−0.61***	0.10*	0.27***	0.36**	0.11	0.69***
LDMC	non-serpentine		−0.53***	0.29***	0.01	0.01	−0.03	−0.45**
	serpentine		−0.39***	0.31***	−0.25***	−0.01	0.05	−0.21*
LT	Non-serpentine			−0.31***	0.01	−0.08	−0.06	−0.23
	serpentine			−0.31***	−0.07	−0.35**	−0.06	−0.32**
LL	non-serpentine				−0.1*	−0.01	−0.07	−0.03
	serpentine				−0.23***	0.13	−0.19	0.23*
LW	non-serpentine					−0.12	−0.16	0.46**
	serpentine					0.26*	0.08	0.01
LNC	non-serpentine						0.41***	0.51***
	serpentine						0.09	0.34**
LCC	non-serpentine							0.11
	serpentine							0.15

***, *P*<0.0001; **, *P*<0.01; *, *P*<0.05. Abbreviations in Table 1.

**Figure 3:**
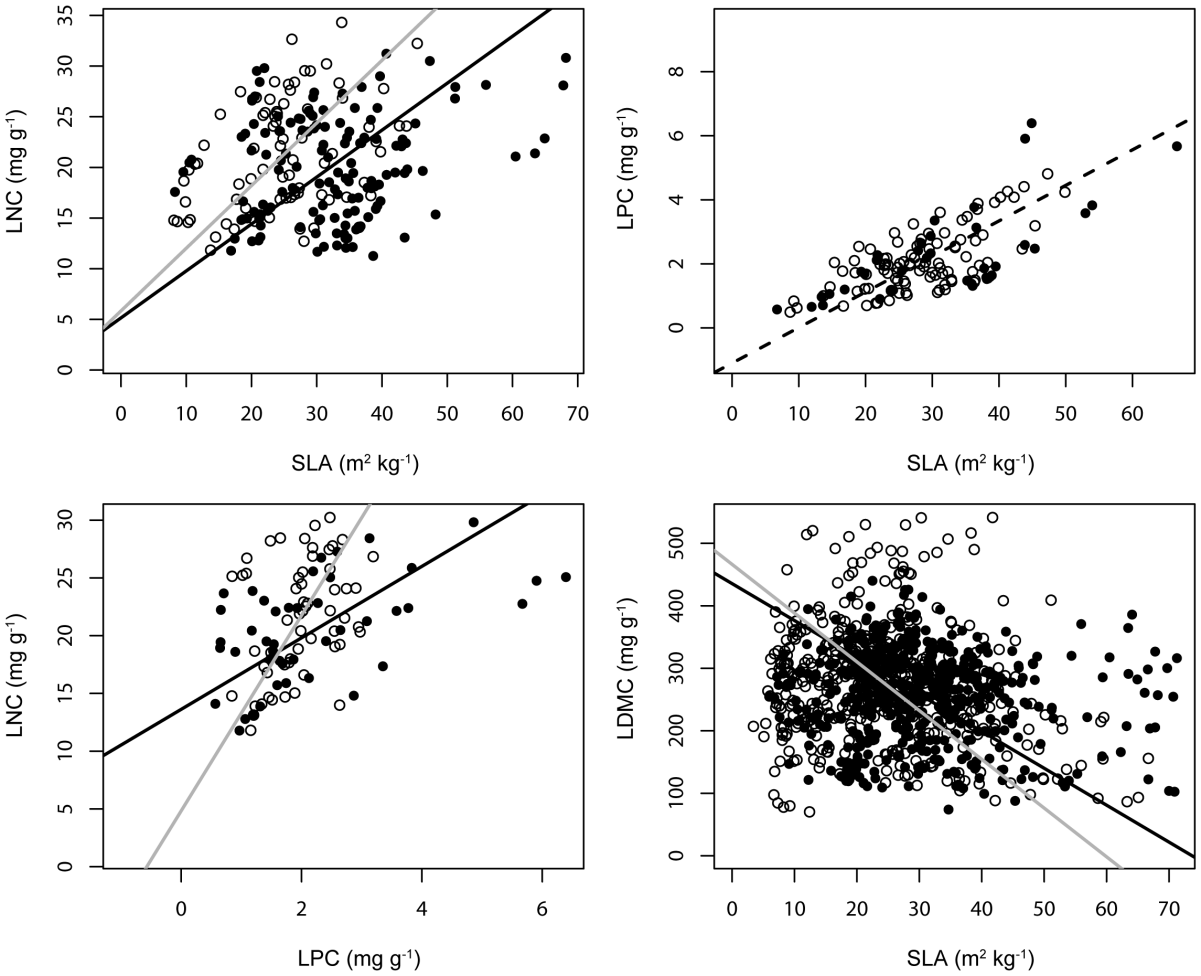
Variation on relationships between traits of the leaf economic spectrum across substrate type differentiation. Non-serpentine substrates are represented by solid circles and black lines, serpentine substrates are represented by open circles and grey lines, while the dashed lines represent the models that coincide.

Standardized major axis (SMA) analysis was used to examine the consistency of four relationships between core ‘leaf economic’ traits across different substrates. Significant differentiations in slopes across different substrates emerged for the relationships SLA vs LNC, LPC vs LNC and SLA vs LDMC (Table 4; Figure 3). The three relationships were significant on both non-serpentine and serpentine substrates (Table 4). The models describing the relationship SLA vs LPC coincided across different substrates (Table 4; Figure 3). The relationship SLA vs LPC was significant across serpentine and non-serpentine substrates (Table 4).

**Table 4:** Results of standardized major axis (SMA) analysis for relationships between traits of the leaf economic spectrum across substrate type differentiation.

	non-serpentine				serpentine				sig. of difference		
	Slope	Intercept	*P*	R^2^	Slope	Intercept	*P*	R^2^	in slope	in elevation	along common slope
SLA vs LNC	0.463	5.149	0.012	0.045	0.618	5.838	0.001	0.131	**0.036**	N.A.	N.A.
SLA vs LPC	0.108	−1.196	<0.001	0.476	0.115	−1.164	<0.001	0.474	0.656	0.198	0.244
LPC vs LNC	3.094	13.62	<0.001	0.256	8.460	4.833	0.009	0.119	**0.001**	N.A.	N.A.
SLA vs LDMC	−5.910	435.5	0.050	0.008	−7.801	466.3	<0.001	0.027	**0.001**	N.A.	N.A.

NA, not applicable.

Bold numbers represent significant differentiations (*P*<0.05) in slope or elevation and/or shift along a common slope.

## Discussion

All measured leaf traits, except LNC, LCC and LPC, varied significantly across the different substrates. SLA, LL and LW presented on average higher values on non-serpentine substrates while species from serpentine substrates showed higher values of LDMC and LT. Species trait values from non-serpentine substrates are associated with a more exploitative strategy. Exploitative species tend to acquire resources rapidly by presenting high values of SLA (low values of LDMC) along with high relative growth and photosynthesis rates [15]. In addition, it is well known that leaf sizes (leaf length and leaf width) are smaller in drier and nutrient poor environments [7], [32] such as serpentine habitats. On the other hand, species from serpentine substrates presented a conservative strategy, investing more resources to structural compounds and thus presenting higher values of LDMC (low values of SLA) and leaf thickness (denser leaves). Considering that leaf thickness has been associated with water storage processes [33], the higher values of this trait on species inhabiting serpentine substrates may relate to the low water-holding capacity of these substrates [34]. Hence with respect to our first question, the transition between the two contrasting environments is associated with changes in traits which are consistent with the leaf economic trade-off [14], [15], [35] and highlight differences in functional strategies followed by the same species at different environments.

In our study, values of LCC did not significantly differ between serpentine and non-serpentine substrates despite the fact that high values of this trait represent investments in structural strength [36]. Grassein et al. [37] suggest that species' strategies are associated not only with plant traits but also with trait plasticity, demonstrating that conservative species exhibit constant values of a structural trait across a resource availability gradient in contrast to a significant range of values presented for this trait by the exploitative species. In our case, it is possible that higher trait plasticity of species from non-serpentine substrates in LCC is responsible for this lack of differentiation across contrasting substrates. In addition, both LNC and LPC were unaffected by the substrate type. It appears that the effect of any potential differentiation in nutrient availability between serpentine and non-serpentine substrates is diluted due to the overall low nutrient availability that characterizes Mediterranean ecosystems [27], [36], [38], [39]. Navas et al. [36] also found no significant variation in leaf nitrogen and phosphorus concentrations across Mediterranean successional stages and attributed this lack of differentiation to the low soil nutrient availability.

The significant species by substrate interactions that emerged for all leaf traits (except LCC; Table 2) indicate the variation in species responses across the different environments. However, the strength of this interaction's effect depends on the trait under consideration. These results are consistent with other studies demonstrating differentiation in species responses to environmental variation [40] and may reinforce studies suggesting that within-species trait variability is not only considerable but also species- and trait-specific [17], [41].

Despite the fact that species differed in their trait responses to substrate types, it seems that at the single-trait level, species responded in the same direction. All the single-trait species' correlations between the two contrasting environments, except for SLA, LNC and LPC, were significant (Figure 1). In other words, with respect to our second question, the species' single-trait responses were generally consistent, conserving the species ranking across the contrasting environments. Consistent rankings of species' trait responses have also been demonstrated across several spatial, temporal, environmental and climatic gradients [42], [43], . On the other hand, the transition from serpentine to non-serpentine substrates caused widely diverse species' multivariate-trait responses that tended to cancel each other out and result in a non-significant mean species shift (Figure 2). Thus, the transition between the two contrasting environments forced the species' traits to respond idiosyncratically. This result reinforces the generalization of the pattern found by Wright and Sutton-Grier [40], who also demonstrated uncoordinated trait variation while studying a different set of species under control conditions. The coordinated leaf trait variation described among global vegetation [15], [46], [47], [48], [49] is not supported in our local scale study between serpentine and non-serpentine habitats.

The significant bivariate correlations that emerged between several leaf traits within both serpentine and non-serpentine substrates followed similar patterns across the two contrasting environments (Table 3). The relationships between traits of the leaf economic spectrum were significant on both substrate types (Table 4). However, the SMA analysis revealed significant slope differentiation for the relationships SLA vs LNC, LPC vs LNC and SLA vs LDMC across the different substrates. Our results indicate that these relationships are probably environment-specific at a local scale and thus are in agreement with other studies presenting either significant differentiation in slopes, in intercepts and/or significant shifts along a common slope for the relationships of the leaf economic spectrum (e.g. [40], [49], [50], [51], [52]). On the other hand, in congruence with the leaf economic spectrum, the relationship between SLA and LPC was identical across the contrasting environments. In response to our third question, although the core ‘leaf economic’ relationships were supported, variations in the strength of these relationships emerged across the different substrates (except SLA vs LPC relationship). Our results demonstrate the dissimilarity of leaf trait coordinated relationships between serpentine and non-serpentine habitats and indicate divergent trait evolution on these edaphically contrasting communities [47].

## Conclusion

In general, we found that leaf trait values varied significantly in response to substrate differentiation. The same set of species followed a conservative strategy on serpentine substrates and an exploitative strategy on non-serpentine ones, in agreement with leaf economic spectrum predictions. However, the considerable within-species trait variability that emerged indicates that at a local scale species may not necessarily be adequately characterized by a unique mean trait value, especially across contrasting environments. Single-trait responses across contrasting substrates were generally consistent while multivariate-trait responses were widely diverse and thus non-predictable. Finally, although the relationships between core ‘leaf economic’ traits were confirmed, the strength of these relationships altered across the different substrates, indicating divergent trait evolution on serpentine and non-serpentine communities.

## Supporting Information

Table S1 Species used on this study.
(DOCX)
