# Supplementary material for: Species Adaptive Strategies and Leaf Economic Relationships across Serpentine and Non-Serpentine Habitats on Lesbos, Eastern Mediterranean
Source: PLoS One. 2014 May 6;9(5):e96034. doi: 10.1371/journal.pone.0096034 (PMC4011732; doi:10.1371/journal.pone.0096034)
Supplement: Table S1 — Species used on this study. (DOCX) [file pone.0096034.s001.docx]

**Supplementary Material**

**Table S1.** Species used on this study.

|  | Family | Genus | Species |
| --- | --- | --- | --- |
|  |  |  |  |
| 1 | Poaceae | *Aegilops* | *biuncialis* |
| 2 | Primulaceae | *Anagallis* | *arvensis* |
| 3 | Poaceae | *Avena* | *barbata* |
| 4 | Asteraceae | *Crepis* | *commutata* |
| 5 | Poaceae | *Cynosurus* | *echinatus* |
| 6 | Poaceae | *Dactylis* | *glomerata* |
| 7 | Apiaceae | *Filago* | *eriocephala* |
| 8 | Brassicaceae | *Hirschfeldia* | *incana* |
| 9 | Poaceae | *Hordeum* | *bulbosum* |
| 10 | Apiaceae | *Lagoecia* | *cuminoides* |
| 11 | Poaceae | *Lolium* | *rigidum* |
| 12 | Plantaginaceae | *Plantago* | *lagopus* |
| 13 | Rosaceae | *Sanguisorba* | *minor* |
| 14 | Apiaceae | *Torilis* | *nodosa* |
| 15 | Poaceae | *Trachynia* | *distachya* |
| 16 | Fabaceae | *Trifolium* | *angustifolium* |
| 17 | Fabaceae | *Trifolium* | *arvensis* |
